# Hybrid Mesoporous Nanoparticles for pH-Actuated Controlled Release

**DOI:** 10.3390/nano9030483

**Published:** 2019-03-26

**Authors:** José L. M. Gonçalves, Carina I. C. Crucho, Sérgio P. C. Alves, Carlos Baleizão, José Paulo S. Farinha

**Affiliations:** Centro de Química Estrutural and CQFM-Institute of Nanoscience and Nanotechnology, Instituto Superior Técnico, Universidade de Lisboa, 1049-001 Lisboa, Portugal; joseluis@tecnico.ulisboa.pt (J.L.M.G.); carina.crucho@tecnico.ulisboa.pt (C.I.C.C.); sergio.c.alves@tecnico.ulisboa.pt (S.P.C.A.)

**Keywords:** smart hybrid nanoparticles, mesoporous silica nanoparticles, RAFT polymerization, pH-responsive, polymer shell, controlled release

## Abstract

Among a variety of inorganic-based nanomaterials, mesoporous silica nanoparticles (MSNs) have several attractive features for application as a delivery system, due to their high surface areas, large pore volumes, uniform and tunable pore sizes, high mechanical stability, and a great diversity of surface functionalization options. We developed novel hybrid MSNs composed of a mesoporous silica nanostructure core and a pH-responsive polymer shell. The polymer shell was prepared by RAFT polymerization of 2-(diisopropylamino)ethyl methacrylate (pKa ~6.5), using a hybrid grafting approach. The hybrid nanoparticles have diameters of ca. 100 nm at pH < 6.5 and ca. 60 nm at pH > 6.5. An excellent control of cargo release is achieved by the combined effect of electrostatic interaction of the cargo with the charged silica and the extended cationic polymer chains at low pH, and the reduction of electrostatic attraction with a simultaneous collapse of the polymer chains to a globular conformation at higher pH. The system presents a very low (almost null) release rate at acidic pH values and a large release rate at basic pH, resulting from the squeezing-out effect of the coil-to-globule transition in the polymer shell.

## 1. Introduction

The development of smart delivery systems has received extensive attention in recent years for application in numerous areas [1]. For most applications these systems should be traceable, accommodate a large payload, and be able to deliver the cargo on-demand at the desired location. Amongst the various materials that have been used as nanocontainers, mesoporous silica nanoparticles (MSNs) offer a number of advantages due to their tunable particle and pore dimensions, large specific surface area and pore volume, good colloidal stability, and the possibility to selectively functionalize the inner pore surface and the external particle surface [2,3,4,5]. In addition, their mechanical stability makes them generally more attractive than nanocapsules [6].

In order to control the release of the cargo from the pores, the outer surface of the particles has been capped with different gatekeepers such as polymers [7,8], copolymers [9,10], DNA fragments [11], and small molecules [12]. In particular, stimuli-responsive polymers have attracted much interest due to their ability to undergo a reversible change between coil and globule conformations [13], in response to stimuli as diverse as light [14], pH [1,14,15], temperature [9,10,16], etc. The ability to control the release of encapsulated active agents [17] is useful in numerous applications, from corrosion protection [18] to the delivery of bioactive species [1,19,20], etc.

Among the different stimuli, pH has attracted special attention because it can be used as an intrinsic trigger in anticorrosion coatings [21], therapeutics [22], etc. Corrosion is a major industrial problem, causing huge financial losses [23,24]. The process causes local pH changes that can be used to trigger the delivery of corrosion inhibitors by pH-controlled release systems [25,26,27,28]. pH-responsive systems can also be very useful in drug delivery applications, because different organs and cell compartments have characteristic pH values, and pH values also change between normal and abnormal tissues. For example, chronic wounds (pH ~5.4–7.4) and most cancer tissues (pH ~5.7–7.8) have extracellular pH values different from those of the corresponding healthy tissues and the bloodstream [13,29,30]. Silica-based nanocontainers are particularly interesting for pH-responsive systems since the charge of the silica structure itself changes with pH [31]. This can be explored to obtain a cooperative effect with the pH-responsive polymer, increasing the efficiency of the cargo release system.

Another important aspect for many applications of nanocarriers for controlled release is their traceability [9,10]. In particular, dye-tagged nanocarriers can be localized by optical techniques. The covalent incorporation of dyes into the silica structure of MSNs using functionalized molecules with alkoxysilane groups is an efficient strategy to avoid dye aggregation and self-quenching effects. This not only results in very high brightness per particle, but also avoids dye leaching and reduces photodegradation of the dyes due to the lower oxygen concentration and diffusivity in the silica structure [32]. Perylenediimide (PDI) derivatives are especially appropriate to prepare such nanoparticles due to their inherent photostability, high brightness, and the possibility to tune the excitation and emission wavelengths in the visible and the near-infrared (NIR) regions [9,10,33,34].

Here, we report a smart hybrid nanocontainer composed by a fluorescent mesoporous silica nanostructured core and a shell of a pH-responsive polymer—poly(2-(diisopropylamino)ethyl methacrylate (pDAEM). This biocompatible polymer is based on a polybase of a tertiary amine methacrylate and has pKa ~6.5 [35], which means that the polymer chains are in an expanded coil conformation at acidic pH values and collapse to a globule conformation in alkaline conditions. The polymer was grown from the MSN surface by reverse addition–fragmentation transfer (RAFT) polymerization to allow control of the shell thickness and homogeneity. A fluorescent dye (PDI derivative) was incorporated to the structure of the core during the MSNs synthesis, to provide traceability. To test the cargo loading and controlled release performance, we used a model fluorescent molecule, sulforhodamine B (SRB). The cargo release is continuously monitored as we modulate the pH of the media, using a method previously developed in our group [10]. The setup avoids the interference of light scattering by the nanoparticles, emission of the cargo still entrapped in the pores, and the intrinsic fluorescence of the MSNs [36,37]. Nanoparticles with different polymer shell sizes were tested, showing promising results for their use as “on-demand” pH-triggered delivery nanocarriers.

## 2. Materials and Methods

### 2.1. Materials

Absolute ethanol (99.9% EtOH, Scharlau), N-cetyltrimethylammonium bromide (CTAB, 99%, Sigma-Aldrich, St. Louis, MO, USA), sodium hydroxide (NaOH, pure EKA Pellets, Bohus, Sweden), hydrochloric acid (HCl, 37%, AnalaR NORMAPUR – VWR), tetraethoxysilane (TEOS, 99%, Sigma-Aldrich, St. Louis, MO, USA), 3-aminopropyl triethoxysilane (APTES, 98%, Sigma-Aldrich, St. Louis, MO, USA), 4-Cyano-4-(phenylcarbonothioylthio)pentanoic acid (CPADB, 97%, Sigma-Aldrich, St. Louis, MO, USA), and *N*-(3-Dimethylaminopropyl)-N′-ethylcarbodiimide (EDC, 98%, Sigma-Aldrich, St. Louis, MO, USA) were used without further purification. Azobisisobutyronitrile (AIBN, 99%, Sigma-Aldrich, St. Louis, MO, USA) was recrystallized twice from methanol before use. Sodium dihydrogen phosphate monohydrate (NaH_2_PO_4_, 98%, Panreac Quimica S.A, Barcelona, Spain), disodium hydrogen phosphate (Na_2_HPO_4_, 99%, Riedel-de-Haen, Seelze, Germany), and sodium hydroxide (NaOH, 98%, Sigma-Aldrich, St. Louis, MO, USA) were used to prepare the phosphate buffer solutions (PBS, 100 mM, pH 5 and pH 9). Toluene, dichloromethane, and triethylamine were distilled over calcium hydride prior to use. Tetrahydrofuran (99% THF, Sigma-Aldrich, St. Louis, MO, USA) and 1,4-dioxane were distilled over sodium prior to use. Sulforhodamine B (SRB, Molecular Probes, Eugene, OR, USA) was used as a model molecule for the release studies in a polypropylene dialysis device with a cellulose membrane (Slide-A-Lyzer Mini Dialysis Devices 10K MWCO, Thermo Fisher Scientific, Waltham, MA, USA). The perylenediimide derivative (PDI) [32] and monomer 2–(diisopropylamino) ethyl methacrylate (DAEM) [38] were synthesized according to the literature. Deionized water from a Millipore system Milli-Q ≥ 18 MΩ cm (with a Millipak membrane filter 0.22 µm) was used in the preparation of solutions and in synthesis.

### 2.2. Synthesis of Mesoporous Silica Nanoparticles (MSN–CTAB)

MSNs were synthesized by a modified sol–gel process. In a polypropylene flask, CTAB (0.500 g) and PDI (6.0 mg) were mixed in THF (5 mL). The mixture was sonicated and left stirring at 40 degrees Celsius until THF was evaporated (approximately 24 h). In a 500-mL polypropylene flask, deionized (DI) water (240 mL) and a NaOH solution (1.7 M, 1.75 mL) were added. The mixture was left stirring until temperature reached 32 degrees Celsius, and afterwards the solid mixture CTAB/PDI was added. After 30 min, TEOS (2.5 mL) was added dropwise, and the solution was left stirring for 3 h. The dispersion was filtered under vacuum, and the solid was washed with a mixture of ethanol and water (50% *v*/*v*). The solid obtained was dried at 50 degrees Celsius overnight and latter under vacuum.

### 2.3. MSNs Amine Functionalization (MSN-NH_2_)

The functionalization of the silica surface with amine groups was performed dispersing 0.6 g of MSN–CTAB in a solution of APTES (0.15 mL) in dry toluene (22 mL). The reaction mixture was kept at 130 degrees Celsius under argon atmosphere for 24 h. The nanoparticles were recovered by centrifugation and washed three times with ethanol (13,600 g with three cycles of 10 min each). The particles (MSN-CTAB-NH_2_) were dried in a ventilated oven for 24 h at 50 degrees Celsius. 

The template was removed resuspending and sonicating (15 min) MSN-CTAB-NH_2_ in an acidic ethanol solution ([HCl] = 0.5 M, 25 mL per 500 mg of particles). The mixture was left under stirring at 50 degrees Celsius for 24 h. MSN-NH_2_ were recovered by centrifugation and washed with a basic ethanol solution (NH_4_OH 25% *v*/*v*) (13,600 g, one cycle of 10 min) and ethanol (13,600 g, five cycles of 10 min each). The solid (MSN-NH_2_) was dried at 50 degrees Celsius for 24 h.

### 2.4. Surface Modification with a RAFT agent (MSN-CTA)

The extracted MSN-NH_2_ were dispersed in dry dichloromethane (23 mL) under argon atmosphere and sonicated for 20 min. The reaction mixture was cooled in an ice bath then CPADB (1 eq. to APTES) and EDC (1.2 eq. to APTES) were added and the mixture was left stirring, under argon atmosphere, at room temperature for 24 h. MSN-CTA were recovered by centrifugation and washed three times with ethanol (13,600 g, three cycles of 10 min each). The product was dried at 50 degrees Celsius for 24 h.

### 2.5. Polymer Grafting to the MSN Surface (MSN-pDAEM)

In a Schlenk flask (A), MSN-CTA nanoparticles (100 mg), and AIBN (0.27 mg) were added under argon atmosphere. In a separate schlenk flask (B), a polymerization reaction was conducted using two times the quantity of CTA present in the Schlenk A ([AIBN]:[CTA] = 1:5). At 3 h (≈50% conversion) the mixture from Schlenk B was transferred to Schlenk A with a cannula under argon and left at 80 degrees Celsius for 24 h. The core–shell nanoparticles (MSN-pDAEM) were recovered by centrifugation and washed three times with ethanol (13,600 g, three cycles of 10 min each). The particles were dried at 50 degrees Celsius overnight and later under vacuum. The polymer present in the supernatant was precipitated with diethyl ether. After, the polymer was dissolved in ethanol and dried under vacuum. We prepared two samples of MSN-pDAEM with different shells sizes, by changing the amount of monomer used in the polymerizations (929 µL of DAEM in MSN-pDAEM55, and 186 µL of DAEM in MSN-pDAEM12).

### 2.6. Loading and Release of SRB

A physical entrapment method was used to load the cargo molecules in the MSN pore structure. In this case, we use SRB as model cargo molecule to test the load/release capability of the hybrid MSN-pDAEM. An ethanolic solution of SRB (3.90 × 10^−3^ M, 500 µL) was added to 1.5 mg of dry MSN-pDAEM, and the dispersion was stirred overnight at room temperature. The dispersion was centrifuged to remove the unloaded SRB, and the nanoparticles were redispersed twice in 1 mL of phosphate buffer (pH 9) and centrifuged. After the last centrifugation, the supernatant was removed and 200 µL of PBS (pH 9) was added to the loaded nanoparticles. The mixture was transferred to the dialysis device and inserted on top of the fluorescence cuvette (previously filled with 3.2 mL of PBS (pH 9)) immediately before the beginning of the experiment [10]. SRB released from the nanoparticles was monitored through the fluorescence intensity of SRB on the bottom compartment of the fluorescence cuvette, while modulating the pH between 5 and 9, with additions of H_2_SO_4_ (1 M, 18 µL) (3600 s, 7920 s, 12,000 s, and 16,560 s) for 6 h. The additions were made in the dialysis device. The supernatants were recovered and used to determine the loading efficiency, by UV–Vis absorption [10]. A control sample was prepared, using MSN without template and no functionalization, designated “bare MSNs”.

### 2.7. Methods

Transmission Electronic Microscopy (TEM): Hitachi transmission electron microscope (Hitachi High-technologies, Tokyo, Japan), model H-8100, with a LaB6 filament (Hitachi High-Technologies Europe GmbH, Krefeld, Germany) complemented with an accelerator voltage of 200 kV and a current of 20 µA. A camera KeenView (Soft Imaging System, Münster, Germany) is incorporated in this equipment, which through iTEM software, allows acquiring TEM images. MSN dispersed in ethanol were prepared and dried on a Formvar carbon coated copper grid 200 mesh (Ted Pella, Redding, CA, USA). Nitrogen Adsorption studies (BET): gas porosimeter (Micromeritics) with an accelerated surface area and porosimetry system. X-ray diffraction: RIGAKU MiniFlex II with an-X ray tube of CuKα (30 kV/15 mA). ^1^H NMR: AMX-400 instrument (Bruker, MA, USA). UV–Vis spectroscopy: UV-660 UV–VIS Spectrophotometer (JASCO International, Tokyo, Japan), supplied with a double monochromator and a photomultiplier detector for higher resolution. pH measurement: bench pH/mV/°C meter pH 1000 L, pHenomenal^®^. Dynamic Light Scattering (DLS): Zetasizer Nano ZS (Malvern, model ZEN3600) equipped with a 4 mW He–Ne solid-state laser operating at 633 nm and backscattered light was detected at 173°. To determine the hydrodynamic diameters of the nanoparticles, the autocorrelation function was analyzed by the CONTIN method. Zeta potentials were calculated from electrophoretic mobility using the Smoluchowski relationship. Disposable folded capillary cells (DTS1070) were used for the measurement of zeta potentials. All measurements were performed in triplicate. Gel Permeation Chromatography (GPC): The system was a Shimadzu (Kyoto, Japan) Prominence consisting of a LC-20AD peristaltic pump, a DGU-20A3R degassing unit and a Rheodyne 7725i injector (injection volume of 50 μL). Three detectors in series were used: a Shimadzu Prominence RF-20A fluorimetric detector, a multiangle static light-scattering (MALS) Wyatt MiniDawn Treos detector, and a Shimadzu RID-10A Refractive Index detector (internal temperature 40.0 °C). The chromatography columns were two Phenolgel analytical columns (Phenolgel^TM^ from Phenomenex, Torrance, CA, USA, 30 cm × 7.8 mm, pore sizes of 10^3^ and 10^4^ Å; column temperature: 23.0 °C) and a Phenolgel linear precolumn from Phenomenex using dry THF as the eluent at a flow rate of 0.8 mL/min. Fluorescence measurements: Horiba-JobinYvon (Kyoto, Japan) Fluorolog-3 spectrofluorometer using a fluorescent cell. Right angle geometry was used in all measurements.

## 3. Results

**Scheme 1:**
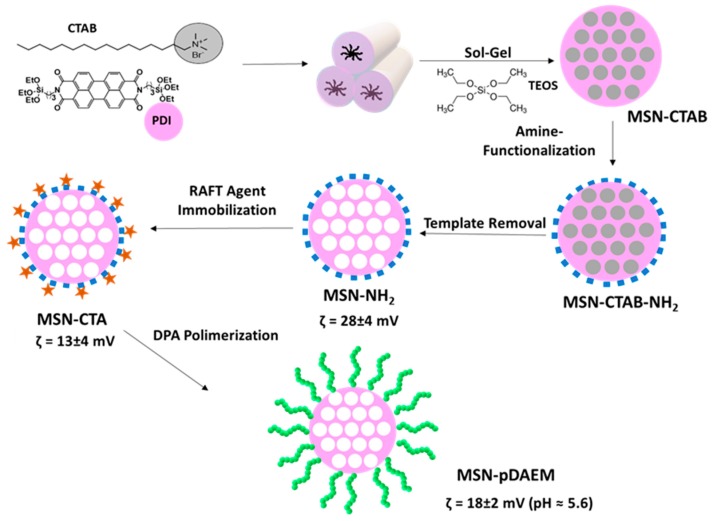
Schematic illustration of the preparation of fluorescent hybrid mesoporous silica nanoparticles (MSNs) with a pH-responsive shell prepared by RAFT. Zeta-potential values measured at pH = 5.6 reflect the change in surface charge upon surface modification.

We prepared a novel pH-responsive control–release system, composed of a mesoporous silica core and a shell of pH-responsive polymer (Scheme 1). The strategy relies on the synthetic versatility of MSNs, which allows the incorporation of a fluorescent dye in the silica network and the external surface modification to incorporate a chain transfer agent, from which pH-responsive polymer chains are grown by RAFT, while keeping the pores free to accommodate the cargo.

**Figure 1:**
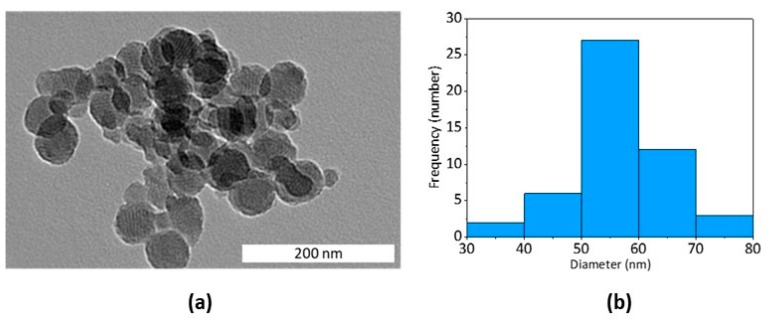
(**a**) TEM image of the MSNs, showing their ordered porous structure. (**b**) Particle diameter distribution obtained by the analysis of 50 particles in TEM images.

The MSNs were prepared by the sol–gel method, with the incorporation of a fluorescent dye (a perylenediimide derivative-PDI) in the silica network. The MSN preparation method is in aqueous media, and the PDI was first adsorbed to the template (CTAB) cylindrical micelles. These micelles aggregate into bundles, and the hydrolysis/condensation of TEOS in basic medium originate the silica network around the CTAB micelles. The obtained nanoparticles have an aligned pore structure visible in the TEM image (Figure 1), with an average particle diameter of (57 ± 9) nm (obtained by the analysis of 50 particles in TEM images). Nitrogen sorption isotherm of the MSNs after template extraction yielded type IV isotherms, typical of mesoporous materials (Figure S1A, ESI). BET analysis gave a surface area of 890 m^2^ g^−1^, and BJH yielded a pore volume of 0.65 cm^3^ g^−1^, with a pore diameter of 2.8 nm (Figure S1A, ESI). Powder X-ray diffraction (PXRD), after template extraction, confirmed the hexagonal arrangement of the mesopores (Figure S1B, ESI) with a diffraction pattern typical of MCM-41 materials.

The silica nanoparticles surface can be easily modified using alkoxysilanes, allowing the covalent immobilization of different molecules. Additionally, it is possible to selectively functionalize the external and the internal surface of the nanoparticles (before and after template extraction, respectively). In order to produce a pH-responsive polymeric shell on the external surface of the MSNs, using RAFT-controlled polymerization, we first anchored a chain transfer agent (CTA) onto the external surface of the MSNs. The MSNs (before template extraction) were modified with APTES to incorporate amine groups that will react with the CTA. The amine concentration, determined by ^1^H NMR [39] (Figure S2, ESI), was 0.84 mmol g^−1^, corresponding to a surface density of 1.7 amine groups per nm^2^. After template removal with an ethanolic acidified solution (mild conditions important to guaranty the integrity of the incorporated PDI), the immobilization of the RAFT agent was performed in the presence of EDC. The amount of immobilized CTA was determined by UV–Vis spectroscopy [32] (Figure S3, ESI), using the spectra of MSN-NH_2_ to subtract the light scattered by the nanoparticles. The RAFT concentration obtained (0.08 mmol g^−1^) corresponds to a surface density of 0.16 molecules per nm^2^; a value chosen to avoid termination reactions in the early stages of the polymerization [40]. The zeta-potential values (Scheme 1) also indicate that the MSNs surface was efficiently modified, with the bare MSNs (without template) showing a negative zeta-potential (−20 ± 5 mV) typical of silica, and the MSN-NH_2_ a positive value (28 ± 4 mV). This value decreases (13 ± 4 mV) upon immobilization of the RAFT agent, indicating a successful reaction [41].

By using RAFT polymerization to grow the polymer shell, we aimed to obtain polymer chains that are monodisperse in size, to achieve a precise control over cargo release. The immediate approach is to grow the polymer chains in a grafting-from process, using only the CTA anchored on the nanoparticle external surface. However, it was reported (and observed by us) that the addition of free CTA in solution results in higher polymer incorporation, since the CTA excess during the polymerization promotes the exchange of oligomeric radicals between the grafted RAFT agents. In addition, this method helps decrease the cross termination of the polymeric radicals [42]. Therefore, we use a hybrid grafting method in which we added free CTA to the polymerization media. This increases the efficiency of polymer grafting, while allowing the analysis of the polymer by using the free chains that remain in the polymerization medium at the end of the synthesis.

The polymerization reaction was performed using different amounts of monomer to obtain polymer shells of two different sizes. The polymer chains resulting from the free CTA added to the reaction were recovered at the end of the polymerization and used to determine the molecular weight (*M_n_*) and size dispersity of the polymer by GPC-MALS (Figure S4A,B, ESI). We obtained *M_n_* = 55 kDa and *M_n_* = 12 kDa (for MSN-pDAEM55 and MSN-pDAEM12 samples, respectively), with size dispersities of *Ð* = 1.04 and *Ð* = 1.18, respectively. The low size dispersity values indicate an excellent control of the polymerization kinetic by the immobilized CTA. The *M_n_* of the polymers was confirmed by UV–Vis spectroscopy (using the CTA moiety absorption), with results very similar to those of GPC-MALS (64 kDa and 13 kDa for MSN-pDAEM55 and MSN-pDAEM12). The small difference between UV-Vis and GPC results is due to the fact that UV–Vis only measures polymer chains initiated by the CTA, while some chains are initiated by AIBN (this can be corrected by taking into account the CTA to initiator ratio). The later method provides a fast and simple way to determine the molecular weight of RAFT polymers, without the need for GPC equipment. The modification of the nanoparticles surface was also confirmed by the increase in the zeta potential due to the presence of positively charged polymeric chains.

The variation of hydrodynamic diameter (*D_H_*), obtained by dynamic light scattering (DLS), also indicates the presence of the polymer shell, further showing that the shell responds to pH variation as intended. For pH values lower than the pKa of the monomer (~6.5) [35], the polymer is in an expanded random coil conformation (*D_H_* = 107 nm for MSN-pDAEM55 and *D_H_* = 75 nm for MSN-pDAEM12), with an hydrodynamic diameter that is significantly larger than that obtained for bare MSN, *D_H_* = 55 nm. For pH values above the pKa of the monomer, the polymer shell collapses into a globular conformation, with sizes that should be similar to that of bare particles (these could not be measured due to the lower colloidal stability of the particles and the weight-averaging of DLS overweighting even a very small amount of aggregates, as observed in the autocorrelation curves, Figure S5, ESI).

To evaluate the performance of our hybrid nanoparticles as a control release system, we used sulforhodamine B (SRB) as model cargo. SRB is water-soluble and has high fluorescence quantum yield, allowing very precise quantification down to nanomolar concentrations. To load the hybrid MSNs with SRB, we placed the nanoparticles (previously dried under vacuum) in contact with a concentrated ethanol solution of SRB. With the polymer chains expanded, the SRB is able to diffuse into the MSN pores. The nanoparticles were then washed with PBS (pH 9) to remove the SRB molecules adsorbed to the external surface of the nanoparticles. At this pH value the polymer chains are collapsed (pH higher than the pKa of the tertiary amine monomer), minimizing the leaching of incorporated SRB from the mesopores. This is the most often used method for the loading of small molecules such as SRB [10]. The amount of SRB loaded into the nanoparticles was determined by UV–Vis absorption, yielding ca. 0.35 mmol/g for MSN-pDAEM55 (thicker polymer shell) and 0.28 mmol/g for MSN-pDAEM12 (thinner polymer shell).

The release of SRB from the nanoparticles was followed in real-time, using a fluorescence cuvette fitted with a top chamber separated from the bottom compartment by a dialysis membrane [10]. The SRB released from the pores diffuses across the membrane, from the top compartment to the bottom one, where the fluorescence intensity of the SRB is monitored. With this setup, we were able to avoid the interference of light scattering by the nanoparticles, and to separate the fluorescence of released SRB from that of SRB in the pores and of PDI in the MSN structure. 

**Figure 2:**
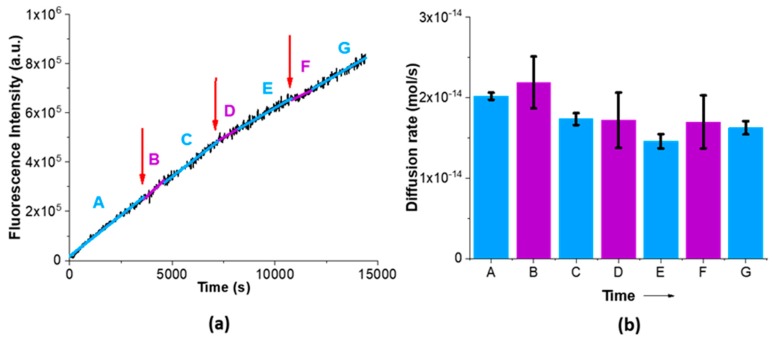
(**a**) Fluorescence intensity of sulforhodamine B (SRB) (solid black line) that diffuses across the dialysis membrane (measured in the bottom compartment of the cuvette, in PBS). The system is followed continuously for 4 h (*λ_exc_* = 566 nm, *λ_em_* = 589 nm). The red arrows indicate acid additions in the top compartment of the cuvette, blue lines (zones A, C, E, and G), and purple lines (zones B, D, and F) indicate basic and acid pH periods in the top compartment, respectively. (**b**) Diffusion rates of free-SRB in solution at basic (blue) and acid (purple) pH—A to G indicate the time intervals shown in (**a**); average number of points for the calculation of each error bar, *n* = 100.

Before the release experiments with the hybrid nanoparticles, we determine the influence of pH in the dialysis device, specifically, how it impacted the diffusion across the membrane. A solution of SRB in PBS (pH 9) was loaded into the top compartment of the fluorescence cuvette and the fluorescence intensity was monitored for 4 h in the bottom compartment, with additions of acid every hour. The pH changes from 9 to 5 in the dialysis device (top chamber) after each addition, while the intervals between the additions ensure that the equilibrium is restored and the pH in the device recovers to ca. pH 9 (Figure S6, ESI). One first conclusion from this control experiment is that the quantum yield of SRB is not affected by pH changes in the 5 to 9 pH range (Figure 2a), as expected [43]. The second conclusion is that the diffusion rates of SRB through the dialysis membrane are not affected by the changes in pH (Figure 2b). In fact, the diffusion rates of SRB obtained at basic and acid pH are almost constant during the experiment, with very similar average values of (17.1 ± 0.7) × 10^−15^ mol/s and (19 ± 3) × 10^−15^ mol/s, respectively. The diffusion rates were calculated using a fluorescence calibration curve for SRB in PBS (Figure S7B, ESI) to determine the amount of SRB.

**Figure 3:**
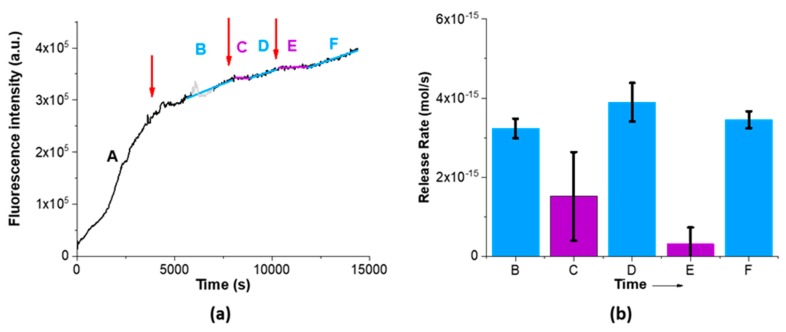
(**a**) Fluorescence intensity of SRB released from SRB-loaded bare MSNs (solid black line) that diffuses across the dialysis membrane (measured in the bottom compartment of the cuvette, in PBS). The system is followed continuously for 4 h (*λ_exc_* = 566 nm and *λ_em_* = 589 nm). The red arrows indicate acid additions in the top compartment of the cuvette, blue lines (zones B, D, and F), and purple lines (zones C and D) indicate basic and acid pH periods in the top compartment, respectively. (**b**) Diffusion rates of SRB released from the bare MSNs at basic (blue) and acid (purple) pH—B to F indicate the time intervals shown in (**a**); average number of points for the calculation of each error bar, *n* = 60. Time interval A corresponds to desorption of SRB remaining at (or close to) the particle surface after the cleaning procedure.

Since the surface charge of silica changes with pH, it is important to evaluate this effect on the release of cargo from the MSNs pores. The isoelectric point of silica is at ca. pH = 2, so that the silanol groups are protonated (positively charged) at pH < 2, and deprotonated (negatively charged) at pH > 2 [24]. Using bare MSNs (no polymer shell) we were able to load 0.28 mmol of SRB per gram of MSN. This is similar to the loading obtained using the hybrid polymeric MSNs (above), meaning that in ethanol the polymer chains in the shell do not hinder the diffusion of SRB molecules into the mesopores in the hybrid nanoparticles. Release experiments were performed in the same conditions as for free-SRB, but using SRB-loaded bare MSNs in the top compartment of the cuvette (Figure 3).

The SRB-loaded MSNs were washed with PBS before the release experiment; however some SRB remains adsorbed on the silica surface, which is the reason for the initial very high slope in Figure 3a (zone A). Addition of acid significantly reduces the slope (zone B in Figure 3a), indicating that the protonation of the silica surface reduces the initial leaking of the adsorbed cargo due to electrostatic interaction. Therefore, we set the beginning of the experiment as the first addition of acid, and zone A was not considered when we calculate the release rates (Figure 3b). Further acid additions led to a stabilization of the fluorescence intensity in the bottom compartment (purple lines in Figure 3a), resulting from a decrease in SRB release from the pores and diffusion across the membrane (zones C and E). This is explained by the partial protonation of the silanol groups at lower pH, which establish electrostatic interactions with SRB, and the formation of hydrogen bonds between the silanol groups and SRB. Mixing of the volume in the top compartment with the (larger) bottom compartment volume increases the pH in the top compartment from pH 5 to the value of the bottom compartment, ca. pH 9. This increase leads to deprotonation of the silanol groups, which results in easier release of SRB from the pores due to the pore/bulk SRB concentration gradient (zones D and F in Figure 3a). In conclusion, we observe that the change in silica surface charge with pH can provide some degree of control over the release of the cargo. The average SRB release rates at basic and acid pH are in this case significantly different, with values of (3.5 ± 0.3) × 10^−15^ mol/s and (0.9 ± 1) × 10^−15^ mol/s, respectively. Although this control is far from the performance we seek, this can be explored to obtain a synergetic effect with the pH-responsive polymer.

**Figure 4:**
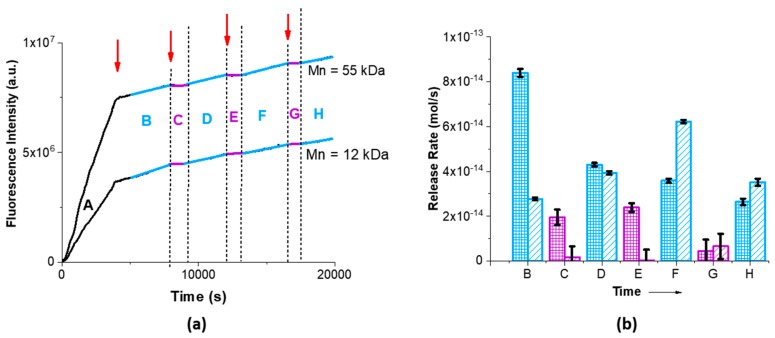
(**a**) Fluorescence intensity of SRB released from SRB-loaded core-shell MSN-pDAEM nanoparticles with polymer chains of *M_n_* = 55 kDa and *M_n_* = 12 kDa, MSN-pDAEM55 and MSN-pDAEM12 respectively, that diffuses across the dialysis membrane (solid black lines, measured in the bottom compartment of the cuvette, in PBS). The system is followed continuously for 5 h (*λ_exc_* = 566 nm, *λ_em_* = 589 nm). The red arrows indicate acid additions in the top compartment of the cuvette, blue lines (zones B, D, F, and H) and purple lines (zones C, E, and G) indicate basic and acid pH periods in the top compartment, respectively. (**b**) Diffusion rates of SRB released from MSN-pDAEM55 (diagonal pattern) and MSN-pDAEM12 (grid pattern) at basic (blue) and acid (purple) pH—B to H indicate the time intervals shown in (**a**) and the rates were calculated using the intervals marked by the traced lines in (**a**); average number of points for the calculation of each error bar, *n* = 70. Time interval A corresponds to desorption of SRB remaining at the particle surface after the cleaning procedure.

To study the release control performance of the hybrid MSN-pDAEM samples, we used the same conditions as for the bare MSN (Figure 4). Again, some SRB remains adsorbed on the nanoparticle surface after cleaning with PBS. The initial very high slope resulting from SRB desorption (Figure 4a, zone A) was not considered when we calculate the release rates (Figure 4b) and we set the beginning of the release experiment as the first addition of acid.

The conformational response of pDAEM to pH variation is related to the protonation/deprotonation of its tertiary amines. At pH lower than the pKa of the polymer (considered close to that of the monomer, pKa = 6.5), the tertiary amine in DAEM is protonated and the polymer chains are in an expanded coil conformation due to charge repulsion, while at pH higher pKa the polymer chains are not charged and adopt a collapsed globule conformation. This change in conformation could hypothetically affect the release of SRB in two opposite ways. If the coil/globule conformations observed at low/high pH act to open/close the pore entrance, the polymer chains act as pore gates and the effect would oppose that observed for silica alone (where release was observed at high pH, Figure 3). On the other hand, if the positive charges of the expanded polymer chains at acid pH interact electrostatically with the negative charges of SRB and retain the cargo in the polymeric shell, release would happen at higher pH where the electrostatic effect is lost and the collapse of the chains can squeeze the cargo out [10]. In the second hypothesis one would expect a synergistic effect with both polymer and silica contributing to retain the cargo at low pH and release it at high pH.

Observation of the release experiment results in Figure 4 clearly shows that a better control is obtained with the addition of the polymer shell, so that the control mechanism should rely on the changes in electrostatic interaction and the squeezing effect observed in previous studies [10]. In fact, the release rates at high pH are much larger than for bare MSNs, while at low pH the cargo is almost completely retained (specially for particles with larger polymer shell, MSN-pDAEM55). One also notes that the fluorescence intensity profiles in Figure 4a are similar for both MSN-pDAEM nanoparticles. After the first acid addition the rate of fluorescent intensity decreases as the remaining SRB interacts with the nanoparticle (zone B in Figure 4a). Subsequent acid additions to the top compartment of the cuvette led to an almost complete suppression of SRB release (zones C, E, and G in Figure 4a). This suppression of SRB release at pH < pKa is due to the protonation of both the polymer chains and the silanol groups in the silica structure, both contributing to retain the negatively charged SRB due to electrostatic interaction. The SRB that diffuses from the pores (as in the base MSNs), is then retained is the shell due to the electrostatic interaction with the polymer positive charges. This retention (zones C, E, and G in Figure 4a) remains, while the pH in the top compartment is lower than the pKa of the polymer. After a certain time, mixing of the volume in the top compartment with the (much larger) bottom compartment volume increases the pH in the top compartment from pH 5 to the value of the bottom compartment, ca. pH 9. This leads to the coil to globule transition in the polymer, which becomes uncharged. The loss of electrostatic interaction and the collapse of the polymer chains expel the SRB that diffuses to the lower compartment (zones D, F and H in Figure 4a).

The release rates (Figure 4b) clearly show a very efficient on:off release mechanism, with MSN-pDAEM55 showing a higher variation of the release rate (1400%) compared to MSN-pDAEM12 (300%) as the pH is modulated (basic/acid cycles). The different between the on and off states is very clearly seen on the variation of the average SRB release rates with pH for each polymer shell. For the smaller polymer shell (MSN-pDAEM12), the values are (47 ± 1) × 10^−15^ mol/s and (16 ± 3) × 10^−15^ mol/s at basic and acid pH, respectively. For the larger polymer shell (MSN-pDAEM55), we obtained (41.1 ± 0.9) × 10^−15^ mol/s and (3 ± 5) × 10^−15^ mol/s at basic and acid pH, respectively. The retention at acid pH depends on the thickness of the shell as expected—larger polymer chains (MSN-pDAEM55) have more charged groups and provide a more efficient barrier against diffusion from the pores at low pH (zero release rate within experimental error), accumulating more cargo, which is expelled at higher pH by a squeezing out effect [9,10]. 

Our results on the release of charged cargo provide new insights on the role of silica materials in pH-responsive systems. In fact, the change in silica charge with pH can have a synergistic effect with the pH responsive polymer, enhancing the control over the release of the cargo. This allowed us to obtain an on:off release system, especially effective for the thicker polymer shell (MSN-pDAEM55). The combination of the electrostatic interaction of the silica and the polymer with the charged cargo at low pH, and the simultaneous disappearance of this interaction and coil to globule collapse of the polymer chain resulted in a system with very low (almost null) release rate at acid pH, and a high release rate at basic pH, larger than that observed for silica alone.

## 4. Conclusions

We prepared a novel nanocarrier for pH-actuated controlled release, based on a mesoporous silica core and a pH-responsive polymer shell. In our proof-of-concept release experiments, we used a model anionic cargo molecule (SRB) while modulating the pH between 5 and 9. By using a measuring cell fitted with a dialysis membrane we are able to follow the release of the cargo as the pH is changed in real-time. Release control results from synergy between the core silica structure and the pH-responsive polymer shell. At pH < pKa ≈ 6.5, the electrostatic attraction and hydrogen bonding of the cargo with the protonated amine groups in the extended polymer shell and the silanol groups in the mesoporous silica core retain the cargo with very high efficiency, resulting in a very low (almost null) release rate. At pH > pKa ≈ 6.5, the electrostatic attraction between the carrier (silica and polymer) and the cargo is strongly reduced and the polymer chain collapses to a globule conformation, expelling the cargo molecule due to a sponge-like squeezing-out of the cargo that results in a release rate larger than that observed for silica alone. The performance of the system is especially high for the thicker polymer shell.

By combining a unique set of structural properties, a fluorescent dye for optical traceability and imaging, and a highly effective pH-actuated on:off release control mechanism, our novel smart hybrid nanocontainers have huge potential for application in numerous areas, such as anticorrosion, drug delivery, active catalysis, etc.

## Supplementary Materials

The following are available online at https://www.mdpi.com/2079-4991/9/3/483/s1. Figure S1. (A) Nitrogen adsorption (solid)-desorption (dot) isotherm for MSNs, and corresponding pore size distribution (inset). (B) Powder X-Ray diffractogram of MSNs, showing the pattern for ordered hexagonal mesopores; Figure S2. Solution ^1^H-NMR of MSN-APTES (at pH = 13), with peaks assigned for the APTES propyl chain, showing the surface modification of the nanoparticles; Figure S3. (a) The amount of RAFT agent at the MSNs surface was calculated by subtracting the light scattering contribution (measured for the unlabeled MSNs, gray curve), from the absorption spectrum of MSN-RAFT (blue curve); Figure S4. GPC-MALS chromatogram of pDAEM55 (A) and pDAEM12 (B). Raw data from the light scattering detector (black curve) and refractive index (red curve). Mw distribution (blue curve); Figure S5. Normalized autocorrelation curves for (A) bare MSN, (B) MSN-pDAEM55, and (C) MSN-pDAEM12 at pH > pKa ~ 6.5 (blue curves) and pH < pKa ~ 6.5 (purple curves). For bare MSNs (A) there is no alteration of in the autocorrelation curves with the change in pH, while for the hybrid MSNs the curves at pH > pKa ~ 6.5 (blue) show a displacement of the autocorrelation curves to larger correlation times, indicative of the increase in the hydrodynamic diameter of the nanoparticles, as well as the appearance of a correlation at larger correlation time which is attributed to nanoparticle flocculation (which prevents reliable inversion of the correlation curves to calculate the hydrodynamic diameter of the nanoparticles at high pH), see Figure S6. Schematic representation of the SRB release from SRB-loaded MSN-pDAEM. The polymer is expanded at low pH values and when the pH rises it collapses. When the polymer is expanded SRB diffuses to the surface and it is only released when the polymer collapses; Figure S7. (AA) Emission spectra (*λ_exc_*= 566 nm) of SRB solutions in PBS (pH = 7) with a known concentration of SRB and (BB) the corresponding calibration curve.
