# Supplementary material for: Hybrid Mesoporous Nanoparticles for pH-Actuated Controlled Release
Source: Nanomaterials (Basel). 2019 Mar 26;9(3):483. doi: 10.3390/nano9030483 (PMC6474099; doi:10.3390/nano9030483)
Supplement: Supplementary file 1 [file nanomaterials-09-00483-s001.pdf]

ELECTRONIC SUPPLEMENTARY INFORMATION

Hybrid Mesoporous Nanoparticles for pH-Actuated Controlled Release

José Gonçalves[1], Carina Crucho[1], Sérgio Alves[1], Carlos Baleizão[1,*] and José Paulo Farinha[1,*]

[1] Centro de Química Estrutural and CQFM-Institute of Nanoscience and Nanotechnology, Instituto Superior Técnico, Universidade de Lisboa, Portugal

***** Correspondence: carlos.baleizao@tecnico.ulisboa.pt (C.B.); farinha@tecnico.ulisboa.pt (J.P.F.)

Contents

(A) MSN physicochemical characterization 2

(B) MSN-APTES NMR characterization 3

(C) Determination of the amount of RAFT agent on the MSNs 4

(D) GPC results for pDAEM55 and pDAEM12 5

(E) Autocorrelation data of DLS measurement 6

(F) Schematic representation of controlled release from SRB-loaded MSN-pDAEM 7

(G) Determination of extinction coefficient of SRB in PBS 8

(A) MSN physicochemical characterization

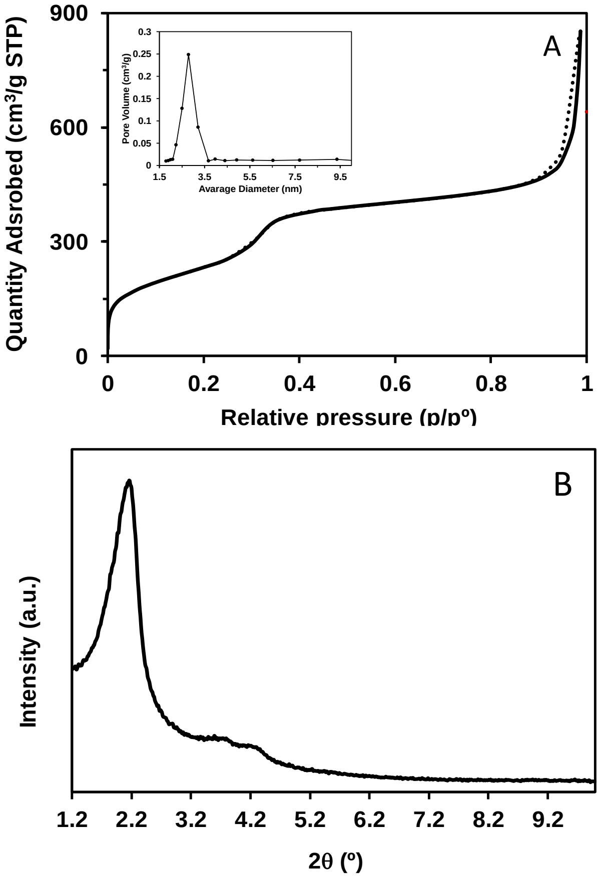

Figure S1. (A) Nitrogen adsorption (solid)-desorption (dot) isotherm for MSNs, and corresponding pore size distribution (inset). (B) Powder X-Ray diffractogram of MSNs, showing the pattern for ordered hexagonal mesopores.

(B) MSN-APTES NMR characterization

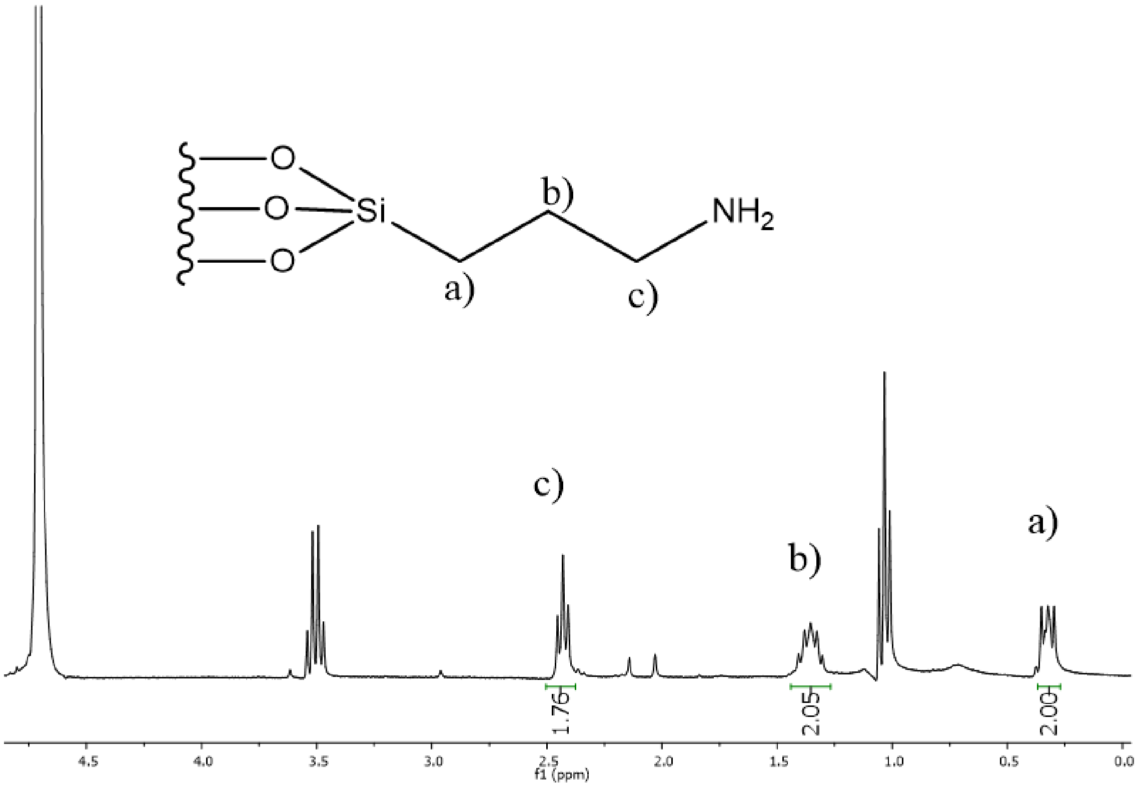

Figure S2. Solution ^{1}H-NMR of MSN-APTES (at pH=13), with peaks assigned for the APTES propyl chain, showing the surface modification of the nanoparticles.

(C) Determination of the amount of RAFT agent on the MSNs

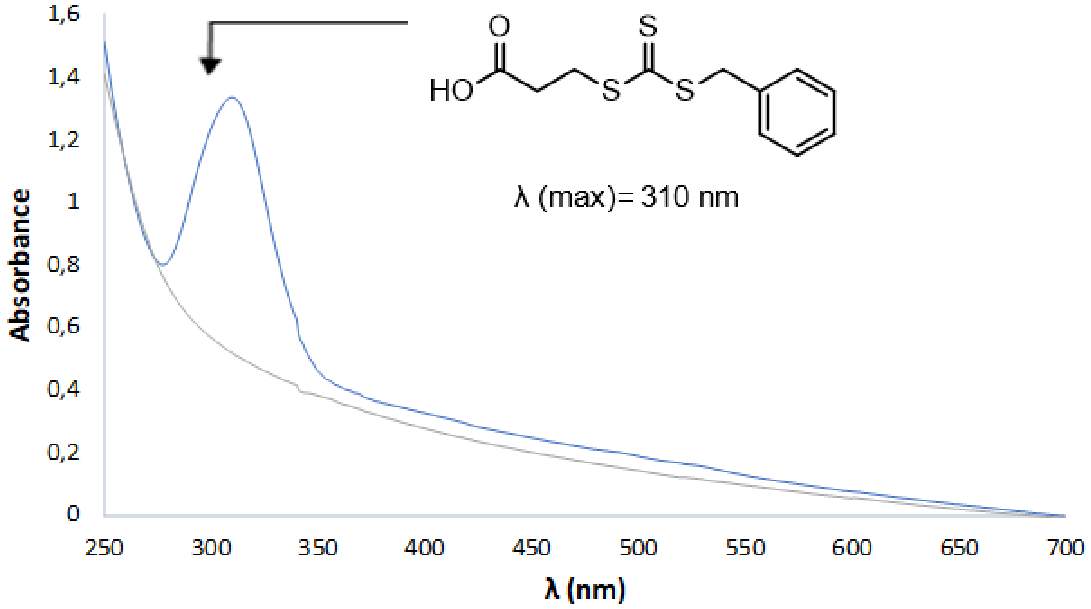

Figure S3. (a) The amount of RAFT agent at the MSNs surface was calculated by subtracting the light scattering contribution (measured for the unlabelled MSNs, grey curve), from the absorption spectrum of MSN-RAFT (blue curve).

(D) GPC results for pDAEM55 and pDAEM12

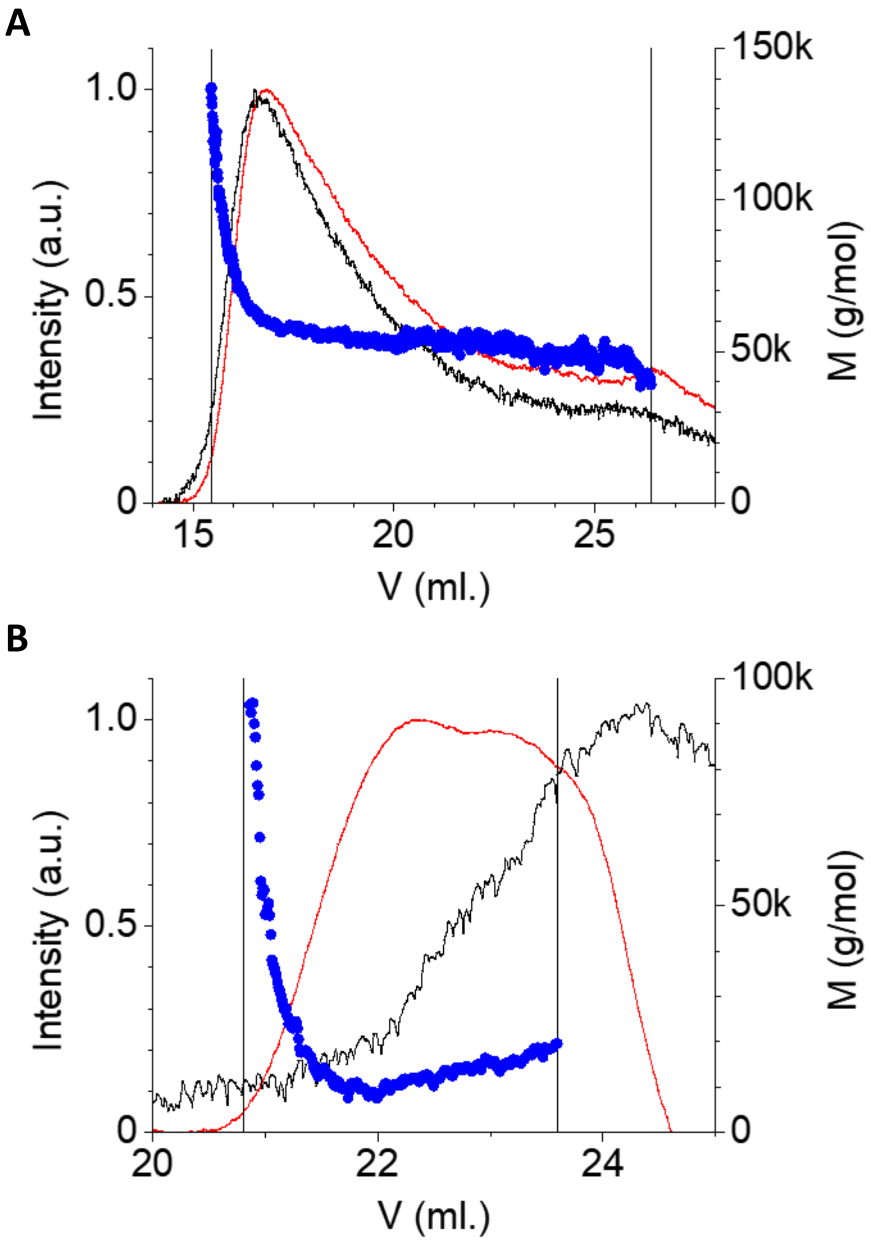

Figure S4. GPC-MALS chromatogram of pDAEM55 (A) and pDAEM12 (B). Raw data from the light scattering detector (black curve) and refractive index (red curve). Mw distribution (blue curve).

(E) Autocorrelation data of DLS measurement

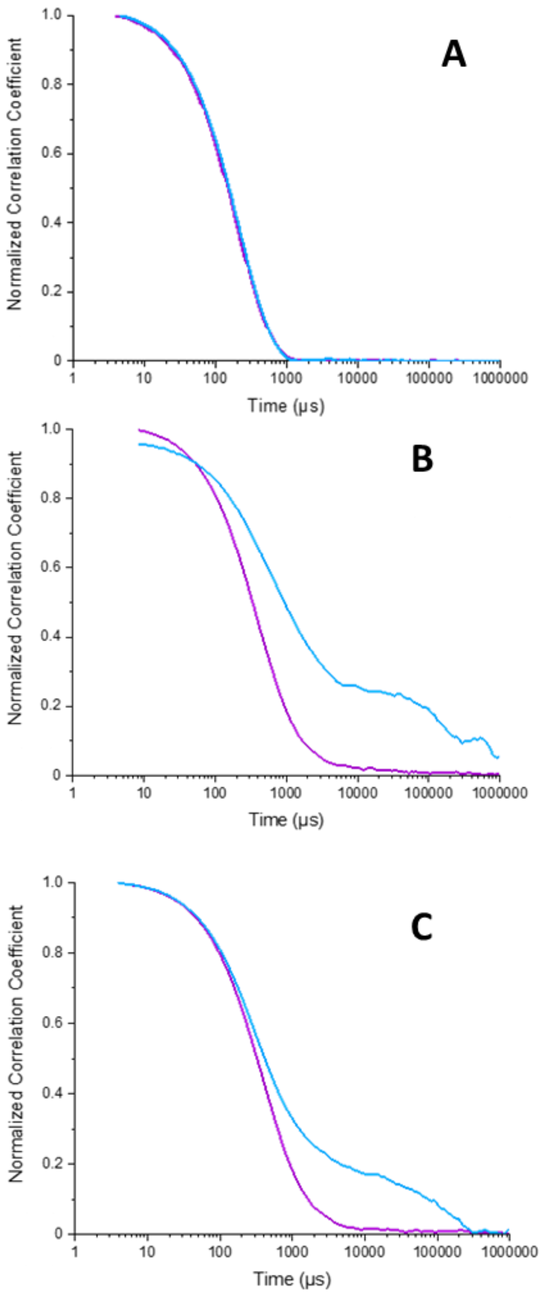

Figure S5. Normalized autocorrelation curves for (A) bare MSN, (B) MSN-pDAEM55, and (C) MSN-pDAEM12 at pH > pKa ~ 6.5 (blue curves) and pH < pKa ~ 6.5 (purple curves). For bare MSNs (A) there is no alteration of in the autocorrelation curves with the change in pH, while for the hybrid MSNs the curves at pH > pKa ~ 6.5 (blue) show a displacement of the autocorrelation curves to larger correlation times, indicative of the increase in the hydrodynamic diameter of the nanoparticles, as well as the appearance of a correlation at larger correlation time which is attributed to nanoparticle flocculation (which prevents reliable inversion of the correlation curves to calculate the hydrodynamic diameter of the nanoparticles at high pH).

(F) Schematic representation of controlled release from SRB-loaded MSN-pDAEM

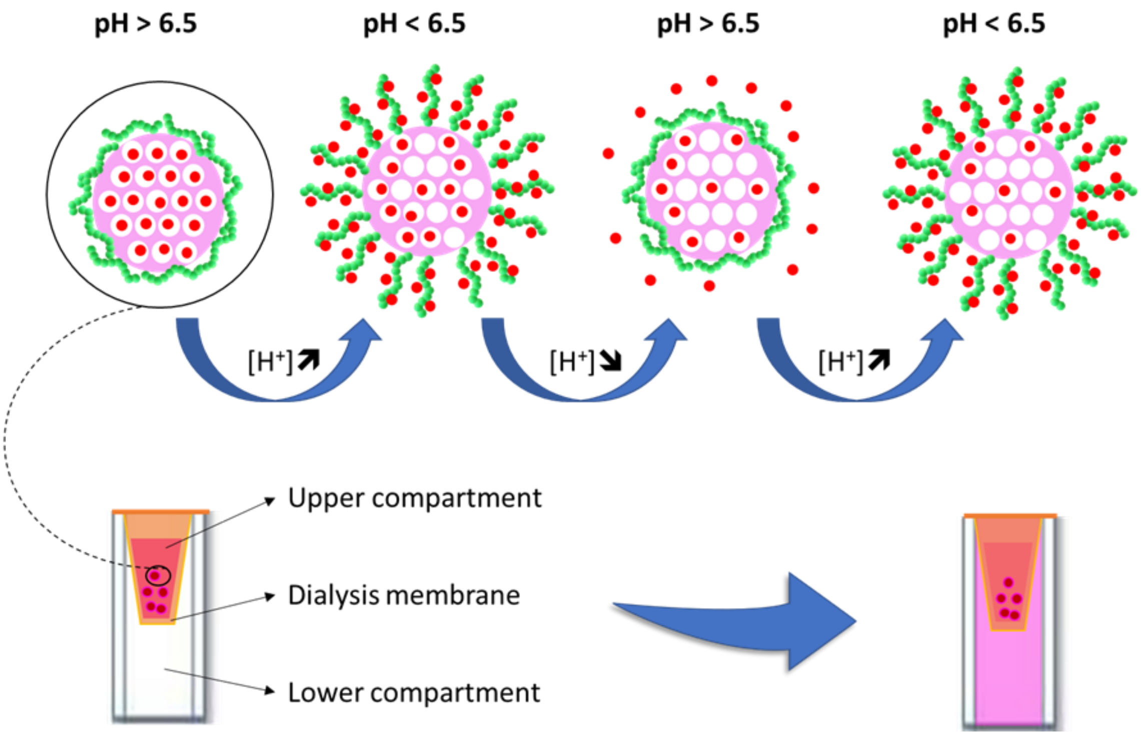

Figure S6. Schematic representation of the SRB (●) release from SRB-loaded MSN-pDAEM. The polymer is expanded at low pH values and when the pH rises it collapses. When the polymer is expanded SRB diffuses to the surface and it is only released when the polymer collapses.

(G) Determination of extinction coefficient of SRB in PBS

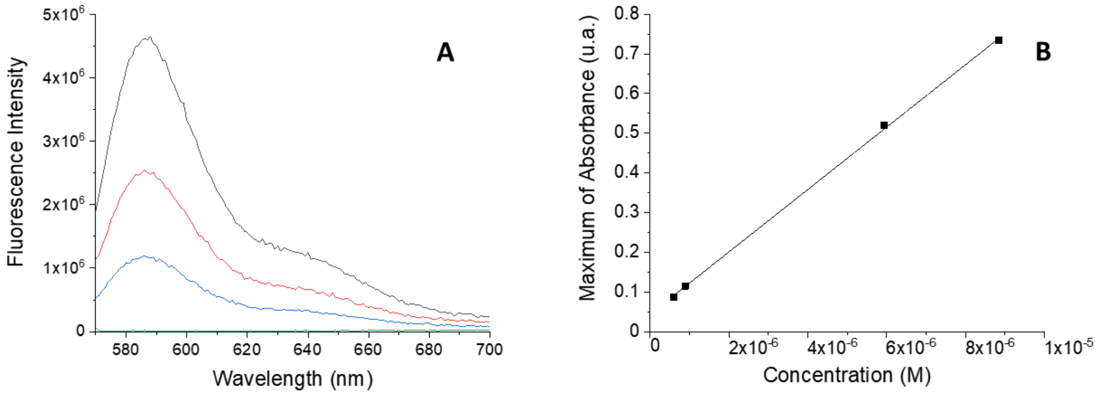

Figure S7. (A) Emission spectra ($\lambda_{excitation} = 566\,nm$) of SRB in PBS (pH = 7) and (B) corresponding calibration curve.
